# Investigating Obstructive Sleep Apnea and Its Treatment on Cognitive Outcomes Using Survey and Real World Data

**DOI:** 10.1093/geroni/igaf122.574

**Published:** 2025-12-31

**Authors:** Christopher Kaufmann, Marcela Blinka, Alden Gross, Jennifer Albrecht, Emerson M Wickwire, Halima Amjad, Atul Malhotra, Adam Spira

**Affiliations:** University of Florida, Gainesville, Florida, United States; Johns Hopkins University, Center on Aging and Health, Baltimore, Maryland, United States; Johns Hopkins Bloomberg School of Public Health, Baltimore, Maryland, United States; University of Maryland, Baltimore, Maryland, United States; University of Maryland, Baltimore, Maryland, United States; Johns Hopkins University School of Medicine, Baltimore, Maryland, United States; University of California, San Diego, San Diego, California, United States; Johns Hopkins Bloomberg School of Public Health, Baltimore, Maryland, United States

## Abstract

With the growing prevalence of Alzheimer’s disease (AD) and AD-related dementias (ADRD), investigators have sought to identify modifiable risk factors that if addressed, can slow cognitive decline and delay AD/ADRD. Obstructive sleep apnea (OSA) affects a billion people worldwide and is linked to serious health consequences, including AD/ADRD. Treatments for OSA (e.g., continuous positive airway pressure [CPAP]), are available and highly effective when used as prescribed. Even so, there is ongoing debate about how these therapies influence cognitive outcomes, largely stemming from the limited number of studies with sufficient follow-up time to account for the extended preclinical phase during which OSA affects patients’ AD/ADRD pathology. In 2022, our team was awarded an R01 from the NIA to investigate the relationship between OSA and cognitive decline and AD/ADRD onset, and whether CPAP can reduce the risk of these negative cognitive outcomes. We are using data from two NIA-funded cohort studies, the Health and Retirement Study (HRS) and the National Health and Aging Trends Study (NHATS), with linked Medicare claims accessed via the newly established NIA LINKAGE Enclave to address these questions. Ultimately, our study leverages nationally representative cohort studies of older adults linked to real-world data to generate insights for dementia prevention. In this presentation, we will a) provide an overview of OSA and its link to cognitive outcomes, b) outline our study’s aims, c) describe our use of HRS and NHATS data linked with Medicare claims, and d) discuss the importance of the NIA LINKAGE Enclave in effectuating our vision.

